# Inhibition of HSV-1 by chemoattracted neutrophils: supernatants of corneal epithelial cells (HCE) and macrophages (THP-1) treated with virus components chemoattract neutrophils (PMN), and supernatants of PMN treated with these conditioned media inhibit viral growth

**DOI:** 10.1007/s00705-012-1306-y

**Published:** 2012-04-12

**Authors:** Kozaburo Hayashi, Laura C. Hooper, Toshiomi Okuno, Yuichiro Takada, John J. Hooks

**Affiliations:** 1 Immunology and Virology Section, Laboratory of Immunology, National Eye Institute, NIH, Bethesda, MD 20892 USA; 2 Department of Microbiology, Hyogo College of Medicine, Hyogo, Japan; 3 Biological Imaging Core, NEI, NIH, Bethesda, USA

## Abstract

The role of PMNs (neutrophils) in corneal herpes was studied using an *in vitro* system. Human corneal cells (HCE) and macrophages (THP-1) infected with HSV-1 or treated with virus components (DNA or virus immune complexes) released chemokines, which attracted PMNs. Highly reactive oxygen species were detected in PMNs. PMNs inhibited HSV when overlaid onto infected HCE cells (50:1). PMNs incubated with the supernatants of HCE cells treated with virus components released H_2_O_2_ and myeloperoxidase. These inhibited virus growth. PMNs released NO and MIG, which may differentiate CD4 T cells to Th1. PMNs participate in innate immune responses, limit virus growth, and initiate immunopathology.

The host responses to corneal herpes simplex virus type-1 (HSV-1) infection elicit vigorous inflammation and neovascularization [1–3]. When HSV-1 is inoculated into the mouse cornea, infectious virus disappears from the afflicted lesions 4 to 5 days postinfection (PI) [4, 5]. How HSV-1 is eliminated and what triggers subsequent immunopathological lesions is still not well understood. Previously, we reported that human corneas contain a high copy number of HSV DNA and deposited HSV immune complexes (HSV-IC) [6]. Human corneal epithelial cells (HCE) and human corneal fibroblasts have been shown to release IL-6 via TLR-3 and -9 [7]. These viral components cause new vessels to be leaky so that leukocytes can pass through them in the afflicted cornea [8]. Here, we attempt to identify chemotactic factors released from corneal cells and macrophages treated with virus components and to study factors released from activated PMNs, which may inhibit virus growth during herpetic corneal infection.

Human corneal epithelial cells (HCEs) were propagated in 6-, 24- or 96-well plates with MEM supplemented with 10 % fetal bovine serum, essential amino acids and antibiotics. THP-1 cells, a human monocyte-macrophage cell line, were cultured in 6- or 24-well plates with 1.0-2.5 × 10^6^ cells/well with RPMI1640 supplemented with 10 % fetal bovine serum, antibiotics and PMA (20 ng/ml). Purified CD16^+^ human PMNs (1 × 10^8^ cells) in RPMI 1640 were purchased from All Cells (Emeryville, CA). Purified human peripheral blood CD4 T cells (HPBT) were purchased from Lonza (Lonza, Walkersville, MD) and cultured in LCM-3 medium supplemented with PHA (1 μg/ml) and rIL-2 (10 ng/ml). HSV-1, Mckrae (5 × 10^7^ PFU/ml) and the MP strain (5 × 10^6^ PFU/ml) were used. They were partially purified by centrifugation at 14,000 rpm for 90 min in a Sorvall SS34 rotor. Some of the partially purified virus was further purified by sucrose density gradient centrifugation (10–60 % w/v) using a Beckman SW28 swing rotor for 1 h at 11,500 rpm. Viral DNA was isolated from the purified virus using a QIAamp UltraSens Virus Kit (QIAGEN, Valencia, CA). Herring sperm DNA purchased from Roche Diagnostics (Indianapolis, IN) was used as a DNA control. To make HSV-1-anti-HSV IgG immune complexes (HSV-ICs), purified HSV-1 (Mckrae and MP strains) were mixed with 5.0 % human γ-globulin (human γ-globulin Cohn fraction II, III: neutralizing titer 1:640; Sigma, Milwaukee, WI) overnight at 4 °C and then centrifuged, and the pellet was washed twice with PBS. HSV-ICs were not infectious when assayed on Vero cell monolayers.

Kinetic and viral-dose-dependent (from m.o.i. = 0.1 to 100.0) release of chemokines (IL-8, Gro-α and a cytokine, GM-CSF, from HSV-treated HCE monolayers) were assayed by ELISA (R&D Systems, Minneapolis, MN). Induction of chemotaxis of PMNs by these supernatants, recombinant IL-8, rGro-α and rGM-CSF and these recombinant proteins plus the corresponding antibodies (R&D Systems) were assayed on 96-well HTS chemotaxis chamber plates (5.0-μm pore size. Corning Life Science, MA) using the alamarBlue (AB) bio-staining method (Invitrogen). Rabbit IgG was used as an antibody control. Wells with serum-free RPMI 1640 served as controls. PMNs (5 × 10^5^ cells/well) were added to the upper inserts and incubated at 37 °C for 2 hours, then the inserts were removed, and 10 μl of AB was added to the bottom wells. After incubation for 18 hours, the number of PMNs in the bottom wells was estimated by absorbance at 570 nm with 600 nm as a reference wavelength. A standard curve was generated with a serial twofold dilution from 1 × 10^6^ to 1.25 × 10^5^ of PMNs.

The activated state of PMNs mixed with supernatants obtained from HCE and/or THP-1 cells treated with HSV-1 components was observed under a fluorescence microscope after incubation for 30 minutes with 10 μM aminophenyl fluorescein (APF, Assay Designs, Ann Arbor, MI) [9]. Release of type 1 interferons, TNF-α and MIG from PMNs treated with recombinant chemokines and rGM-CSF, supernatants of HCE or THP-1 cells treated with viral components was assayed by ELISA (interferon-α [PBL Interferon Source, Piscataway, NJ], interferon-β [FUJIREBIO, Tokyo] and TNF-α [R&D Systems]). For the assay of MIG, equal numbers of PMNs and peripheral CD4 T cells were mixed with the supernatants obtained from virus-component-treated HCE cells. After incubation for 24 hours, supernatants were assayed by ELISA (R&D Systems). PMNs (1 × 10^6^ cells/well) were mixed with supernatants of HCE and/or THP-1 cells treated with HSV-1 components, and the amount of H_2_O_2_ and myeloperoxidase (MPO) released from the PMNs was measured using a Cayman H_2_O_2_ assay kit (Cayman Chemical Co. Ann Arbor, MI) and a human MPO enzyme immunometric assay kit (Assay Design, Ann Arbor, MI). NO release from the treated PMNs was assayed using a total NO/Nitrite/Nitrate assay kit (R&D Systems), which converts the nitrate into nitrite using nitrate reductase. Endogenous nitrite concentrations were subtracted from the values obtained after nitrate reductase treatment.

To measure the viral inhibitory activities of H_2_O_2_ and MPO, HSV-1 (100 PFU/0.1 ml of Mckrae and/or MP strain) was mixed with serially twofold diluted H_2_O_2_ (3,500 to 438 ng/ml) or MPO (25.0 to 3.12 ng/ml) for one hour at 37 °C. After the incubation, the virus titer was estimated. Vero cells pretreated with 3,500 to 438 ng/ml of H_2_O_2_ were used as a control for the cytotoxicity of H_2_O_2_. Experiments were repeated three times, and the results were statistically analyzed by Student’s *t*-test.

After HSV-1 infection, release of IL-8, Gro-α and GM-CSF from HCE cells increased as early as 2 hours PI. IL-8 and Gro-α levels plateaued at 8 hours PI, from 0 to 257.8 ± 38.5 pg/ml and from 0 to 23.0 ± 5.8 pg/ml, respectively. The amount of GM-CSF released was 83.0 ± 11.4 pg/ml at 2 hours PI and reached 453.0 ± 56.2 pg/ml at 10 hours PI. Uninfected HCE did not release IL-8 or Gro-α, while a small amount of GM-CSF (73.5 ± 1.40) was released spontaneously.

Supernatants obtained from cells treated with the MP strain and its components were more potent in their ability to attract PMNs than those obtained from cells treated with the Mckrae strain, and therefore, the results obtained using the MP strain are described. Supernatants obtained from HCE and THP-1 cells infected with the MP strain (m.o.i. = 1.0) and/or treated with its components attracted more PMNs than the control medium except, supernatant obtained from THP-1 cells infected with the live MP strain. When the parametric number of chemoattracted PMNs with supernatants of MP-infected, MP DNA-transfected and MP-IC-treated HCE and /or THP-1 cells was set to 100, supernatants obtained from untreated HCE and THP-1 cells or cells treated with normal rabbit IgG were 0.5 to 4 (p < 0.01). When supernatants were mixed with anti-IL-8 and anti-GM-CSF, the chemotactic activity was reduced from 48 to 0.5 (p < 0.05). When PMNs were incubated with the Mckrae and/or MP strain, cell-free and cell-associated infectious viruses became undetectable at 48 hours PI. When PMNs were overlaid onto HSV-infected HCE cell monolayers (m.o.i. = 10.0 for Mckrae and 1.0 for MP) with a PMN:HCE ratio of 50:1 for 24 hours, virus growth was suppressed; the infectious titer of the Mckrae strain was reduced on average from 1.0 × 10^5^ PFU to 2.3 × 10^2^ PFU/ml and that of the MP strain from 4.0 × 10^4^ PFU to 2.0 × 10^2^ PFU/ml. Supernatants of PMNs mixed with supernatants obtained from Mckrae- or MP-infected or virus-component-treated HCE or THP-1 cells inhibited HSV growth, although the inhibitory activities were weak, ranging from 1/3 to 1/8 of HSV growth compared to cells treated with control supernatant of PMNs. TNF-α was released, from 8 to 25 pg/ml, from PMNs incubated with supernatants of HSV-infected, HSV-DNA- and MP-IC-treated HCE cells and 80 pg/ml from MP-IC-treated THP-1 cells. However, TNF-α was not inhibitory to the virus at these concentrations. Interferon-α, -β and -γ were not released from PMNs mixed with treated HCE and THP-1 cell supernatants. The concentration of MIG released from PMNs plus peripheral CD4 T cells mixed with supernatants of HCE cells infected with Mckrae, transfected with Mckrae DNA, or treated with Mckrae IC ranged from 2,500 to 3,000 pg/ml. HCE cells transfected with herring DNA did not induce MIG in PMNs and peripheral CD4 T cells. H_2_O_2_ was released from PMNs mixed with the supernatants of HCE cells infected with Mckrae and MP, transfected with MP DNA and treated with HSV-IC (Fig. 1a). Release of H_2_O_2_ from PMNs was also seen when supernatants of THP-1 cells were infected with Mckrae, transfected with HSV DNA, or treated with MP-IC. Growth of the Mckrae and MP strains was inhibited in a dose-dependent manner by H_2_O_2_ (Fig. 1b and c). Control Vero cells pretreated with H_2_O_2_ (from 438 ng to 3,000 ng/ml) supported Mckrae strain growth similar to untreated Vero cells. Therefore, H_2_O_2_ inhibited HSV-1 directly. Slightly, though not significantly, more MPO was detectable in PMNs when they were treated with the supernatants of virus-treated HCE cells (Fig. 2a). When HSV-1 was mixed with MPO at 37 °C for one hour, the virus titers decreased with increasing concentrations of MPO (p < 0.01, Fig. 2b and c). PMNs released from 280 μmol to 430 μmol/ml of NO into the supernatant when they were mixed with HCE or THP-1 cell supernatants treated with HSVDNA and HSV-IC (Fig. 3). These levels of NO were significantly higher than those obtained from the untreated HCE supernatant control (p < 0.01). NO does not directly inhibit viral growth at the range of concentrations obtained from treated HCE.

**Fig. 1:**
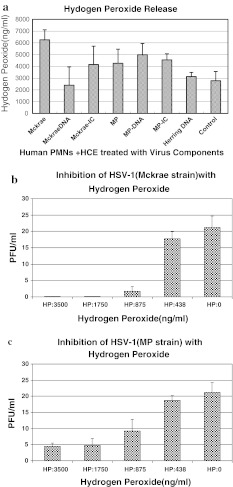
(**a**) Supernatants of HCE cells that had been infected with the Mckrae or MP strain or treated with virus components were mixed with PMNs at 37 °C for 24 hours. The amount of H_2_O_2_ in the supernatants was measured using a Cayman H_2_O_2_ assay kit. Supernatants obtained from Mckrae or MP-infected, MP-DNA and HSV-IC-treated PMNs released more than 4,000 ng H_2_O_2_/ml. (**b and c**) The Mckrae (**b**) and MP (**c**) strains were mixed with serially twofold-diluted H_2_O_2_, incubated at 37 °C for one hour, and then added to the Vero cell monolayers. After adsorption for two hours, the monolayers were washed with PBS and overlaid with complete medium supplemented with 2 % human γ-globulin (anti-HSV neutralizing antibody titer = 1:640). Viral plaques were counted at 48 hours after inoculation. Untreated viruses served as controls. Growth of both strains was clearly inhibited at an H_2_O_2_ concentration above 876 ng/ml (p < 0.01)

**Fig. 2:**
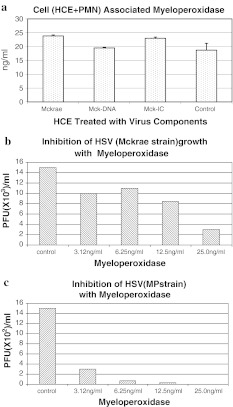
(**a**) MPO was assayed using a human MPO immunometric assay kit (Assay Design). MPO was constitutively cell-associated in PMNs, but slightly more MPO was detectable when PMNs were mixed with the supernatants of virus-treated HCE cells. (**b and c**) When Mckrae (**b**) and MP (**c**) strain of HSV were treated with MPO from 3.12 to 25.0 ng/ml at 37 °C for one hour, growth of both strains was inhibited (Mckrae at 25.0 ng/ml and MP strain, p < 0.01)

**Fig. 3:**
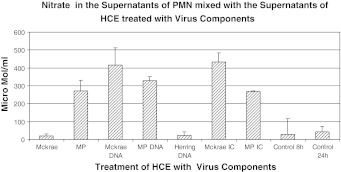
Supernatants obtained from PMNs that had been mixed with HCE supernatants treated with HSV components was deproteinized using 10,000-MW-cutoff filters (R&D systems), and the total nitrite oxide (NO) in the samples was measured using an NO/Nitrite/Nitrate assay kit (R&D). The amount of NO in the PMN supernatants mixed with HCE supernatants treated with HSV DNA and HSV-IC was significantly higher, ranging from 280 to 430 μmol/ml, than in those from untreated HCE cells or the herring DNA control (p < 0.01)

In the local chemokine milieu, PMNs are activated and held at the afflicted site [10]. Following murine corneal inoculation, HSV-1 was cleared after initial exponential growth for 4–5 days [4]. This elimination of active virus growth may be explained at least in part by the innate immune system and the accumulated PMNs [4–6, 11]. In our *in vitro* model system, PMNs were activated, as evidenced by the production of hROS [9], inhibited HSV spread, and did not support HSV growth. These data agree with the previous reports indicating that PMNs initiate virus clearance [4–6]. PMNs release various mediators such as TNF-a, H_2_O_2_, MPO and NO. TNF-α has been suggested to inhibit the virus [11, 12], but in our study, it was not directly inhibitory. H_2_O_2_ and MPO inhibited virus growth within the range of concentrations obtained from the treated and activated PMNs. In addition to H_2_O_2_ and/or MPO, phagocytosis by PMNs was one of the major inhibitory factors, because at this early period of infection, the effects of anti-HSV antibody and T-cell-mediated immunity were only at the very beginning. Corneal herpes occurs most commonly as a reactivated virus infection, and therefore PMNs can recognize antibody-coated virus particles and infected cells with Fc receptor and inhibit virus growth and spread by ADCC [13]. In mice, the chronic phase of infection follows with strong stromal opacity and neovascularization. These are pathognomonic and characteristic of HSK. In these situations, accumulation of viral DNA and deposition of HSV-IC are well documented [6]. PMNs infiltrate the lesions and cause dense corneal haze. A previous report suggested the importance of NO in the defense against corneal herpes [14]. Interestingly, a low concentration of NO is one of the contributing factors for the differentiation of naïve peripheral CD4 T cells into Th1 T cells [15]. Nitrate ranging from 280-430 μ/ml was detected in the supernatant of PMNs mixed with HCE supernatant treated with virus components (Fig. 3). This range of NO concentration may induce naïve CD4 T cells to differentiate to Th1 cells [15]. We added peripheral CD4 T cells to the supernatants obtained from the treated HCE cells plus PMN. However the amount of IFN-γ released from CD4 T cells mixed with the PMN supernatant obtained from untreated HCE cells was not significantly different from that obtained from CD4T cells treated with HCE cells plus PMNs. Neutrophils recruited to the site are induced to produce other factors such as MIG, which is also known to induce and accumulate CD4^+^ Th1 cells [16]. When PMNs and CD4 T cells were mixed and incubated with HCE supernatants treated with HSV components, 2,500 to 3,000 pg/ml of MIG was released. These local environments may contribute to peripheral naïve CD4 T cell differentiation to Th1, but further study of this problem has to be done in the future.
